# Using co-design to develop interventions to address health literacy needs in a hospitalised population

**DOI:** 10.1186/s12913-018-3801-7

**Published:** 2018-12-20

**Authors:** Rebecca L. Jessup, Richard H. Osborne, Rachelle Buchbinder, Alison Beauchamp

**Affiliations:** 1 Monash Department of Clinical Epidemiology, Cabrini Institute, Melbourne, Australia; 2 Department of Epidemiology and Preventive Medicine, Monash University, Melbourne, Australia; 3 Northern Health, Melbourne, Australia; 4 Health Systems Improvement Unit, Deakin University, Melbourne, Australia; 5 Department of Medicine-Western Health, The University of Melbourne, 176 Furlong Road, St Albans, Victoria Australia; 6 Australian Institute for Musculoskeletal Science (AIMSS), The University of Melbourne and Western Health, 176 Furlong Road, St Albans, Victoria Australia

**Keywords:** Health literacy, Interventions, Co-design, Solutions

## Abstract

**Background:**

Health literacy describes the cognitive and social skills which determine the motivation and ability of individuals to gain access to, understand and use information in ways which promote and maintain good health. Suboptimal health literacy is common and is believed to impact up to 60% of Australians. Co-design is a participatory approach to the development of interventions that brings together to staff and patients to design local solutions to local problems. The aim of this study is to describe a staff and patient co-design process that will lead to the development of health literacy interventions in response to identified health literacy needs of hospital patients.

**Methods:**

A mixed methods, two-step sequential explanatory design. Step 1: hospitalised patients surveyed and data analysed using hierarchical cluster analysis to establish health literacy profiles. Step 2: clusters presented as vignettes to patients and clinicians to co-design interventions to address needs.

**Results:**

Eight health literacy clusters were identified from surveys. Seven patients attended two patient workshops and 23 staff attended two staff workshops. Three key themes were identified: organisational, provider-patient, and patient self-care. Within these, five sub-themes emerged: “Good quality communication during hospital stay”, “Social support for health”, “A good discharge”, “Care across the continuum” and “Accessing quality information when home”. Fifteen potential interventions were produced, including changes to message design and delivery, staff training in assessing for understanding, social support to improve understanding, improving communication consistency across the care continuum, and strategic dissemination of web-based resources.

**Conclusion:**

This study identified fifteen strategies to address health literacy needs of a hospital population. Implementation and evaluation will identify sets of strategies that have the maximum patient, clinician and organisational benefit. This approach allows for the development of locally-driven, contextually-appropriate interventions to address health literacy needs.

**Electronic supplementary material:**

The online version of this article (10.1186/s12913-018-3801-7) contains supplementary material, which is available to authorized users.

## Background

Health literacy describes ‘the cognitive and social skills which determine the motivation and ability of individuals to gain access to, understand and use information in ways which promote and maintain good health’ [1]. Although the health literacy field is less than 30 years old, it is founded in century old bioethical principles of patient autonomy, non-maleficence, beneficence, and justice [2]. These principles assume that health care organisations and clinicians deliver care that is sensitive to a patient’s ability to act intentionally, with understanding. An adequate level of health literacy is required for an individual to be able to provide informed consent, and to understand all the information and support they require to appropriately manage acute illness and chronic conditions. The provision of high quality health care in hospitals requires that organisations and clinicians recognise, and can be responsive to, individual differences in health literacy.

In the field of health literacy to date, most research has focused on reading, comprehension and numeracy skills (functional health literacy). The instruments that measure functional health literacy have provided much of our understanding of the prevalence of low health literacy in communities, and the impact an individual’s health literacy has on their health outcomes. Population-based studies assessing functional health literacy have found prevalence rates of low health literacy in the USA to be around 36% [3], and approximately 60% in Australian and Canada [4, 5].

However, measuring only functional health literacy overlooks the complex interaction of contextual factors, cultural and personal values, social resources and individual motivations that influence a person’s ability to understand and act upon information related to their health. The result has been that, while we know that people with low functional health literacy have poorer knowledge about their chronic disease [6–9], lower self-management and medication adherence [10, 11], poorer disease outcomes [7, 9, 12], greater use of hospital services [13–15], and increased mortality [16, 17], we have little understanding of how individual, social and cultural contexts influence an individual’s health literacy. There has been little exploration of the role health professionals and organisations play in providing services that accommodate people with different health literacy needs [1, 18]. Additionally, most studies investigating the effect of interventions to improve health literacy have focused only on modifying the readability and comprehensibility of written health information [16].

The Health Literacy Questionnaire (HLQ) is a nine-domain, multidimensional self-report health literacy instrument that provides insight into an individual’s competencies, supports and experiences with health practitioners and services [19]. In doing so, the HLQ provides an opportunity not only for clinicians to better understand their patient’s health literacy needs, including their social support for health, cultural preferences, personal values and motivations, but for organisations to understand and make efforts to address areas of health literacy need across the communities they serve. One method that organisations can use to develop interventions to address the needs of their users is through co-design.

Co-design is a participatory approach to the development of interventions that brings together staff and patient experience to design local solutions to local problems [20]. This approach means that solutions are produced with an understanding of the local context, and that the end result meets all stakeholder needs. The use of co-design harnesses the collective creativity of staff and managers in the system as well as the users of the system, ensuring local ownership. Solutions designed in this way are more likely to be acceptable to both providers and end users, and therefore adopted and sustained [21]. This study aimed to use a co-design process to create ideas for health literacy interventions, led by patients and staff in response to identified health literacy needs of hospital patients.

## Methods

**Fig. 1:**
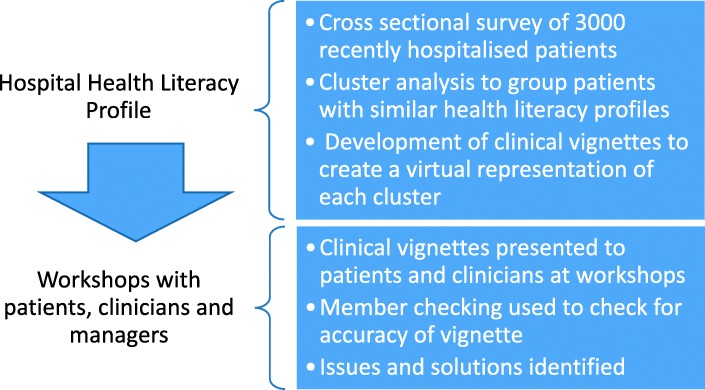
Two phase sequential explanatory design employed to co-create health literacy interventions for hospitalised patients

**Fig. 2:**
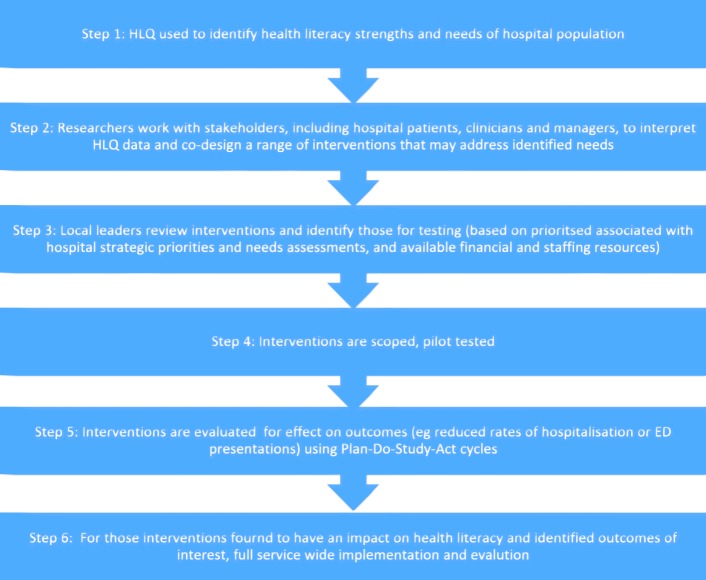
Six step Ophelia (OPtimising HEalth LIteracy and Access) process in the hospital setting (adapted from Batterham et al., [22])

A mixed methods, two-step sequential explanatory design was employed for this study (Fig. 1), based on the Ophelia (OPtimising Health LIteracy and Access) process [22]. Ophelia is a rigorous, evidence based process for identifying, implementing and evaluating health literacy interventions in organisations. It was developed and trialled in the primary health (community health services) setting [23]. The Ophelia process involves six key steps (Fig. 2) underpinned by three evidence-based, complex system approaches to intervention development: intervention mapping, quality improvement collaboratives, and realist synthesis [22]. The research reported in this article focuses on the results from step 2 of the Ophelia process, with a brief description of the results of step 1 (used to inform step 2 and described previously [24]).

### Step 1

The cross-sectional survey of hospitalised patients using the HLQ has been described in detail previously [19]. The survey was conducted at a public hospital located in an area of high socioeconomic disadvantage in Melbourne Australia. The population serviced by the hospital has lower levels of educational attainment, a higher proportion of migrants, and higher rates of unemployment compared with state averages [20], and where health literacy has been found to be lower than at other hospitals [21]. The HLQ is a self-report measure of health literacy that consists of nine conceptually distinct domains across 44 items: 1. *Feeling understood and supported by health care providers*; 2. *Having sufficient information to manage health*; 3. *Actively managing my health*; 4. *Social support for health*; 5. *Active appraisal of health information*; 6. *Ability to actively engage with healthcare providers*; 7. *Navigating the healthcare system*; 8. *Ability to find good health information*; and 9. *Understand health information well enough to know what to do* [22]. The survey has been validated for use in a number of settings [25–28]. Each domain is an independent construct.

Cluster analysis was used to identify and group participants with similar profiles of health literacy scores across the nine HLQ domains [19, 29]. Specifically, the procedure classifies data into mutually exclusive groups based on similarities in patterns of distribution across variables [30]. Clusters were presented as means (SD) for each domain score in each cluster, and accompanied by information about sociodemographic distributions across the cluster. The method for choosing the number of clusters for analysis has been previously described [22], and was guided by an aim to minimise the variance within each domain of each cluster (e.g., if the SD is > 0.6 for one or more of the domains it may indicate that there are still significant subgroups within the cluster); and ensuring that clusters represent different patterns of needs and strengths across the nine domains of the HLQ. Eight clusters were chosen for this study (Table 1). This number provided a clinically meaningful distribution of strengths and needs and was the solution that most closely met our requirements of low variation within the cluster (SD < 0.6) (Cluster solutions available as Additional File 1). Hierarchical cluster analysis of the survey data was performed using Ward’s method for linkage using SPSS Version 22 [31].

**Table 1:** Health literacy eight cluster solution

Number of individuals in cluster	17	11	32	34	61	132	31	53
Average of age	62	73	64	70	55	64	62	65
Number of females	10	3	17	15	36	55	18	27
Number of participants whose first language was not English	8	9	17	22	17	43	7	14
Average ED presentations in 12 months	0.76	0.27	0.81	0.56	0.35	0.41	0.33	1.87
Average number of hospital admissions 12 months	1.35	1.00	1.59	1.38	1.08	1.25	1.43	1.72
Average of LOS for index admission	8.06	2.00	2.91	3.06	2.84	6.44	3.20	3.74
Mean of Health Provider Support	1.90	2.91	2.83	3.18	2.78	3.16	3.46	3.83
Mean of Having Sufficient Information	1.90	3.02	2.43	3.03	2.69	2.98	3.18	3.73
Mean of Actively Managing Health	2.02	3.11	2.63	2.85	2.57	3.03	2.82	3.67
Mean of Social Support for Health	1.81	3.20	2.69	3.21	2.94	3.11	3.39	3.72
Mean of Appraisal of Health Information	1.94	2.87	2.43	2.70	2.58	2.91	2.75	3.49
Mean of Active Engagement	2.47	2.04	2.98	3.46	3.56	4.06	4.70	4.55
Mean of Navigating the Health System	2.25	2.18	2.62	3.38	3.48	3.86	4.44	4.33
Mean of Finding Health Information	2.05	2.11	2.69	2.91	3.50	3.82	4.34	4.30
Mean of Understanding Health Information	2.67	1.95	3.36	3.04	3.87	4.05	4.63	4.52
Standard deviation of Health Provider Support	0.74	0.17	0.32	0.32	0.49	0.31	0.45	0.31
Standard deviation of Having Sufficient Information	0.58	0.31	0.36	0.23	0.35	0.24	0.28	0.29
Standard deviation of Actively Managing Health	0.71	0.23	0.46	0.28	0.42	0.26	0.31	0.32
Standard deviation of Social Support for Health	0.53	0.32	0.42	0.35	0.40	0.40	0.35	0.29
Standard deviation of Appraisal of Health Information	0.54	0.33	0.48	0.38	0.41	0.35	0.33	0.37
Standard deviation of Active Engagement	0.92	0.32	0.43	0.55	0.49	0.26	0.27	0.50
Standard deviation of Navigating the Health System	0.72	0.38	0.44	0.52	0.36	0.31	0.37	0.52
Standard deviation of Finding Health Information	0.71	0.58	0.47	0.55	0.43	0.40	0.37	0.52
Standard deviation of Understanding Health Information	0.92	0.51	0.56	0.56	0.39	0.37	0.33	0.48

**Fig. 3:**
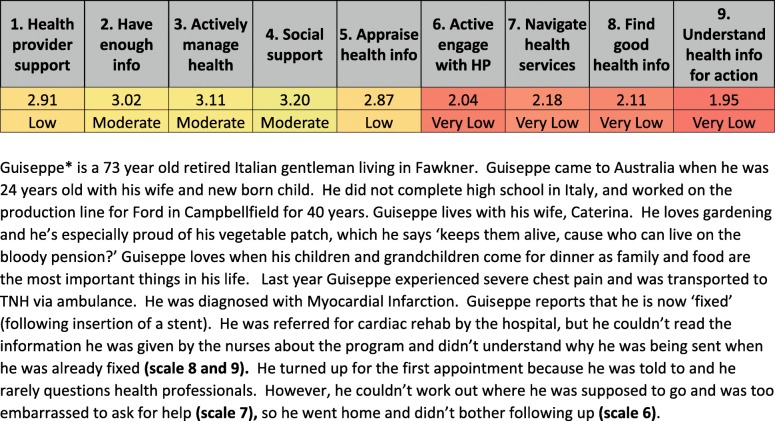
Cluster analysis distribution of scores and associated patient vignette

Clusters were made relevant and user friendly for patients and hospital clinicians and managers through the writing of patient stories (clinical vignettes) – illustrations of how a typical person within each cluster might be impacted by living with that health literacy profile (Fig. 3). Each cluster corresponded to one single vignette. Vignettes were written by the lead author who was a clinician at the health service (and so was familiar with the types of patients attending for care), with member checks made with two health service clinicians as well as all other lead authors to determine the accuracy of the interpretation.

### Step 2

Patient participants were recruited from individuals who had responded to the step 1 survey. Purposive critical case sampling [32] was used to identify twelve respondents (six for each workshop) with a range of health literacy profiles and demographic/clinical backgrounds, to ensure representative groups of patients were included. We contacted a total of 21 participants by phone until 12 consented to participate. We then followed up by providing a copy of the information and consent form in the mail and asked the participant to bring this with them to the workshop. We also phoned each participant the day before the workshop to confirm attendance. We based the number of invited participants on recommended numbers (based on the scientific literature) for non-commercial focus groups, which recommend 5–8 participants [33]. Numbers larger than this may not allow all participants to actively participate and small or ‘mini’ focus groups are considered to be more comfortable for participants [33]. The patients selected for the workshop were selected to represent the spread of health literacy capability (i.e., lower, mixed and higher, with over-sampling of people in the low health literacy clusters), socio-demographic characteristics (age, sex), chronic disease and hospital use profiles who were representative of the step 1 survey respondents and the population serviced by the index hospital [34]. Participants were not paid, but expenses were reimbursed. Each patient workshop ran for two hours.

Purposeful critical case sampling was also used to identify potential participants among clinicians and managers. An ‘all of staff’ email was sent by the Chief Executive Officer seeking expressions of interest from staff to participate in the workshops. From those staff who responded, we sought 23 staff (12 for each workshop) who would represent a broad cross-section of front line clinicians and managers from the wards and outpatient departments and who would therefore be likely to recognise the narratives in the vignettes. In areas where we had low numbers of staff responses, we asked managers from these wards/departments to identify local leaders and directly approach them to participate in the workshops, as they would be influential in adopting identified interventions. To create efficiencies around the time required for staff to attend the workshops, attendees were sent the participant information and consent forms to bring with them to the workshop and provided with pre-reading consisting of a summary of the purpose and results of step 1, the aims of the workshops, and the vignettes for discussion in the workshop. Each clinician workshops ran for 45 min.

The first workshop was facilitated by an experienced researcher who had previously run more than 20 Ophelia workshops (AB). This was used as a training exercise, with the lead author learning the process (RJ). The lead author then facilitated the remaining three workshops, with support from at least one other experienced author who was in attendance (AB and RB). Eight vignettes were designed, with five presented at each workshop. Vignettes used in each workshop were selected in part by the profile of workshop participants and to ensure that all vignettes were presented at least once to staff and patients. Each workshop was run as a single group (all participants together, all of the time), with each participant invited to contribute their thoughts on the seeding questions for each vignette. Workshops were recorded and transcribed with the permission of participants.

Within each workshop, seeding questions were used to test the accuracy of the clinical vignettes in representing ‘usual’ types of patients presenting for care, and to guide discussions in a consistent and systematic way. The questions were slightly different across the groups:

#### Patients

1. Do you recognise the person in this story as someone who could be from your community?
2. What do you think this person’s main issues are?
3. What do you think we could do to improve care for this person?

#### Clinicians and managers

1. Do you recognise the person in this story as someone who could be a patient at this hospital?
2. What could you do to support this person in understanding and managing their condition?
3. What could your organisation do to improve systems and services if there were many people like this in the service?

At the end of each workshop, the unique ideas created in each group were collated and presented back to participants to assess the interpretive accuracy of data collected (initial respondent validation) [35]. These data were then coded across three theoretical themes using von Wagner’s [36] framework of health literacy and health actions, and Paasche-Orlow & Wolf’s [37] evidence-based review of the causal pathways that link health literacy to health outcomes. These three theoretical themes are:

1. Interventions at the organisational, process policy level to address systems access and use;
2. Interventions at the provider-patient interface; and
3. Interventions for patient self-care to address health literacy and disease management.

Thematic analysis is an iterative and reflective process. As we wanted to ensure our conclusions accurately reflected the content of the workshops, two review authors (RJ and AB) separately coded all the workshop data using an iterative constant comparative method [38]. The process involved three stages – initial coding, focused coding to reduce overlap and redundancy of coding, and finally, theoretical coding using the themes identified above [39]. Two cross-checks to compare emerging themes was performed to assess for the accuracy of inferences at each stage. Where a difference was found, the author was asked to demonstrate from the raw data how their interpretation was reached until agreement was found. Where agreement was not found, a third reviewer (RHO) was consulted. A pragmatic approach was adopted when drawing final inferences from this study, with a focus on the development of useful knowledge directly related to health literacy for the index hospital setting. Final inferences were verified by both subject specialists (RB and RHO). Most importantly, and consistent with the co-design process, the data was then referred back to those who attended the workshops (final respondent validation).

## Results

The survey element of step 1 has been presented previously [24]. In brief, 384 patients completed questionnaires. Mean age was 64 years (range 22–96) and 49% were female. Eight separate health literacy clusters were identified (Table 1).

Seven patients (60% attendance rate) attended the two patient workshops (Table 2), three attended the first workshop and four attended the second. Of the patient participants, the median age was 74 years (range 65–85), 2 were female, 43 were in the lowest health literacy cluster, 4 had not completed secondary education, 4 reported an income less than $30,000 and 4 reported having three or more chronic conditions. There was a total of 23 staff participants (96% attendance rate), including 15 nurses (managers, chronic disease specialists, educators, emergency triage, ward and home based), 2 renal dialysis technicians, an Aboriginal and Torres Strait Islander liaison officer, 2 patient experience co-ordinators, a transcultural and language services co-ordinator, and a pharmacist.

**Table 2:** Patient participant demographic and clinical characteristics

Descriptor	*n*=7
Age (years) : median (range)	74 (65 – 86)
Sex (female)	2
Completed secondary education	4
Household income less than $30,000	4
Diabetes	2
Arthritis	4
Heart condition	3
Back pain	3
Stroke	3
Cancer	1
Anxiety/ Depression	4
≥3 chronic conditions	4
Length of hospital stay in days: median (range)	2 (1 – 18)
Combined number of admissions in previous 12 months (median, range)	26 (1, 1 – 12)
Number of ED presentations in previous 12 months	5 (0, 0 – 2)

Across the workshops, a total of 171 ideas to improve the care and services for the personas described in the vignettes’ (intervention ideas) were generated (range 26 to 61 per workshop). Thematic saturation was reached across both patient and staff workshops. Fifteen general strategies across five themes emerged from the analysis and were then grouped across theoretical themes (Table 3). These are presented below, organised according to intervention points across the patient journey from hospital to home.

**Table 3:** Workshop thematic analysis

				Issue/ solution raised in:	
Theoretical theme	Emergent theme	Issue	Solutions	Patient workshop	Staff workshop
Organisation	A good discharge	Follow up appointment letters are too generic and sometimes arrive too late	Appointments provided at discharge, with written explanation of purpose. Translators provided when first language other than English.	✔	✔
	Care across the continuum	Poor continuity of care – hospital does not communicate with GP or external health providers following discharge	Phone call to GP to go over care plan, identify follow up appointments required with GP and with hospital providers.		✔
		Inconsistent messages about management of condition across the care continuum	Shared management of patient using guidelines developed in partnership.		✔
		Outpatient hospital appointments are often too short to allow patients time to ask questions they need to manage their health	Shared medical record (e-health record)	✔	✔
			Follow-up outpatient appointments long enough for patients to ask questions		✔
		Need method to identify patients who don’t have a regular GP, or a good relationship with GP		✔	✔
Patient – Provider interface	Good quality communication during hospital stay	English as a second language not consistently managed well – harder to check for understanding	Request carer/ support person attend appointments and discharge.	✔	✔
			Dedicated time with interpreter to go over key discharge points.	✔	
			Follow up group therapy provided in different languages (where possible) with other patients who speak same language.	✔	✔
		Too much information provided during hospital stay	Use teach back to check for understanding.	✔	
		No one discusses with patient what format they want to receive information in.	Use teach back to check for understanding. Use different methods for teaching (including brochures, videos pictures, demonstration, verbal instruction).		✔
		Health professionals do not check if a patient understands them.	Teach back become part of organisation wide teaching and training for all clinicians.	✔	
		Health professionals use medical terminology when explaining information to patients	Teach back become part of organisation wide teaching and training for all clinicians.	✔	
	A good discharge	Information overload at point of discharge	Use teach back to check for understanding.		✔
			Develop a good care plan that patient is involved in with that has good information sources. This might include links to reliable internet sources. You then need get him to a point where you are certain he understands it.	✔	✔
			Discharge summary explained directly to the patient with a copy provided for them to keep for themselves.	✔	✔
	Social support for health	Patients may not be able to take in everything they are told – a second pair of ears may be helpful	Request carer/ support person attend appointments and discharge.	✔	
		Patient’s need social support for motivation – group therapy with cultural and language groups	Follow up group therapy provided in different languages (where possible) and/ or with other patients from same cultural group.	✔	✔
		Need to identify patients who are socially vulnerable	Identify patients without support networks and link them in to social	✔	✔
Patient self-care	Accessing quality information when home	Both clinicians and patients may rely on the internet for information that is not necessarily reliable	Hospital provide links to reliable sources of information on the web on its website.		✔
			Links to reliable information provided as part of patient care plan.		✔
		Clinicians not always aware of information available in different languages	Hospital communications office provide information in different languages for different presentations		✔

### Good quality communication during hospital stay

Clinicians and patients identified that five main requirements for ensuring communication with patients is of ‘good quality’ were:

1. Developing rapport with the patient so that a relationship of trust is established.
  *“Maybe health care providers can be trained in being more sensitive in talking to patients. [The hospital could run] workshops and create guides for training.”* Clinician, Workshop 2
2. Identifying the patient’s preferred format for receiving information. Where appropriate, have the patient demonstrate relevant self-management skills.
  *“What sort of format would he [the patient] like information in? Work out the best way to engage him?”* Clinician, Workshop 4
  *“Monitor his log books, [get him to] show us how he calculates and administers his insulin [while he is still in hospital].”* Clinician, Workshop 4
3. Use plain language – avoid medical ‘jargon’.
*“Too much jargon – she’s only had basic education – she needs things put out plainly.” Patient, Workshop 3*
4. Use a mechanism to check for understanding.
*“[To determine] whether [a patient] can follow instructions, get them to explain to you what the issue is and what they need to be doing to deal with it. Not really about education - it’s also about where they are in terms of their other life factors.” Clinician, Workshop 4*
*“Can we give them some sort of testing to see if they have got it [the information]?” Patient, Workshop 1*
5. Avoid overloading the patient – deliver information in bite size pieces across their hospital stay.
*“It’s important that in every contact we just try and educate in bits and pieces. Each point of contact with coaching adds up.” Clinician Workshop 4*

### Social support for health

Participants in the workshop identified that social support provides several advantages to patients trying to process complex information. This was regarded as being of particular importance for patients from culturally and linguistically diverse communities, for whom the communication task is more complex. Three overall strategies were suggested:

6) Have a family member or friend attend key appointments.
*“I reckon what he needs is someone to go with him, whose got a bit a nouse, can read what needs to be read and is prepared to spend some time with him and give him what he needs.” Patient, Workshop 1.*
*“He needs something better than an interpreter because they’re there and gone in a flash. He needs someone to go with him. That’s all he needs.” Patient, Workshop 3*
7) Group physical therapy and education sessions for people going through the same health experience, allows for sharing of information, resources, and strategies from the ‘lived experience’.
*“Talking with other people who have similar problems to yourself, sharing your grizzles and problems.” Patient, Workshop 1*
*“[For outpatient group physical therapy, e.g., cardiac rehabilitation] try and group patients with similar background/ same language for interpretation but also for building social networks with people from same background.” Clinician, Workshop 4*
8) Identifying patients who do not have a ‘support person’ (socially isolated), and providing them with a case manager.
*“If the health service can look at a particular patient and see that ‘this guy’s needs are more than what we can give him as far as help, he needs…’, they can perhaps contact his son or daughter and ask them to come to the hospital with him to help him through it.” Patient, Workshop 3*
*“Somehow the system has to pick up that this person doesn’t quite comprehend and put a minder with them or at least contact them once a month ‘just remember Bill, next week you’ve got this appointment’.” Patient, Workshop 3*

### A good discharge

Participants perceived that most of the information patients require to manage their care at home is provided at the point of discharge, and that it is delivered in an inconsistent manner. The following strategy was suggested:

9) Written information (possibly with pictures where appropriate) should be provided as part of discharge. This should be discussed with the patient, in the presence of a carer and/or interpreter where required.
*“You get a discharge summary to give to your GP, but in a sealed envelope and not discussed, and follow up appointments are posted out but often too late. Should be given while in hospital.” Patient, Workshop 1*
*“I reckon a piece of paper with it all written out....Instead they rattle off all these things and you say right oh and you get out the door and you’ve forgotten what the bloody hell they said …. if you had a piece of paper with it written out in big print that everybody could read, it wouldn’t hurt.” Patient, Workshop 3*
*“Should be sent home with a written plan as to what to do when she gets out of bed, for each step in the day – make it really simple.” Patient, Workshop 1*

### Care across the continuum

Many participants perceived that the communication between the hospital and the patient and GP was poor. Letters relating to post-discharge follow up appointments were too generic and provided no information about the purpose of the appointment - only the date, time, location and speciality. Further, due to delays in postage and handling, sometimes these letters were received *after* the appointment date. Follow up appointments were perceived to be so short that they did not allow for proper understanding of information or questions. In addition, participants felt that there was little to no communication between the hospital and their GPs. Participants suggested the following strategies:

10) Hospital follow up appointment dates should be provided at or before discharge, with a written explanation of purpose. Translators should be provided on request. When patients do not attend follow up appointments, they should be contacted by phone.
  *“The outpatient letters are very general and don’t have enough specific information, they could be more user friendly, less jargon so people know where they are going and for what purpose.”* Clinician, Workshop 2
  *“There are other issues where people get inconsistent information about appointments from in hospital and after discharge.”* Patient, Workshop 3
  *“If people miss 2 or 3 outpatient appointments they are discharged from the service. This is often a big barrier ...did anyone follow him up to say why didn’t you attend appointment?”* Clinician, Workshop 4.
11) The hospital should contact the GP directly to discuss outcomes of the patient’s hospital stay and any follow up appointments required.
  *“As an organisation, shouldn’t we be contacting the GP to say this patient needs to see you within 7 days of discharge? Sometimes discharge summaries don’t get sent to the GP.”* Clinician, Workshop 2
12) A shared medical record between the hospital and GP would be ideal, as well as shared use of disease management guidelines so that information is consistent between hospital and GP.
  *“We need electronic medical records to improve communication between providers (e-health).”* Patient, Workshop 1
  *“For patients to trust what health care providers are telling them patients need consistent information, the same story across all providers.”* Clinician, Workshop 4
  *“[We need to have] care pathways so all health care providers know what the pathway is and how to deliver it.”* Clinician, Workshop 4
13) Follow up appointments should be long enough to allow patients to ask questions.
  *“The time consultants spend with patients is very small. Even making lists doesn’t work, as soon as they hit the chair in the office they are out the door again.”* Clinician, Workshop 2

### Accessing quality information when home

Many participants reported a lack of trust in the information provided on the internet. This was felt to be a barrier for patients having information to manage their health. Participants identified several options for providing patients with information following discharge:

14) Provide links to trusted sources of information through the hospital’s website.
  *“Maybe the [hospital] communications office can put up something on their site, FAQs, reputable sites etc.”* Clinician, Workshop 2
15) Provide appropriate brochures and pamphlets, in a patient’s first language, during the hospital stay and at discharge. The discharge plan could also provide credible website addresses.
*“Develop a good care plan that he’s involved with that has good information sources. This might include links to reliable internet sources.” Clinician Workshop 4*

## Discussion

This study identified five key themes that impact on a hospitalised patient’s health literacy; “Good quality communication during hospital stay”, “Social support for health”, “A good discharge”, “Care across the continuum” and “Accessing quality information when home”. Across these themes, 15 strategies were identified to address health literacy needs in individuals and populations. These strategies included changes to the way health information messages are designed and delivered, training programs for staff on how to educate with sensitivity and assess for understanding, using social support for health as a tool to improve understanding, improving communication between hospitals and primary care providers to ensure consistency of information and follow up of care, and strategic communication of quality information online.

These strategies were derived using the Ophelia process to profile patients at a public hospital [18], and then to engage patients and clinicians in the co-design of health literacy interventions. The cross-sectional survey provided rich, context specific health literacy data and was presented in a user-friendly way through a patient story to illustrate what representative patient groups look like, including their specific health literacy profiles (strengths and weaknesses). This resulted in the design of locally-relevant commentary about patients and their health literacy needs and the development of interventions to service these needs that are more likely to be acceptable to both the clinicians and patients. Using the Ophelia process resolves the disconnect between what organisations, clinicians and patients value, and the co-design approach allows for the development of interventions that clearly address identified health literacy needs for this specific local population.

Cluster analysis provides a unique method for applying a non-linear approach to assessing individual and population health literacy. By non-linear we mean that the focus is moved away from specific ‘cut points’ that classify a patient as either ‘low’ or ‘adequate’ health literacy based on specific scores (e.g. a = x, b = x), and focuses instead on meaningful data about the health literacy strengths and needs of individuals within a ‘cluster’. In practice, this might mean that a group of individuals might score really low for ‘*understanding health information well enough to know what to do’* but identify as having very high ‘*social support for health’.* Scoring really well in a category such as social support or health provider support, might mediate low scores in another such as understanding or finding health information. When we label patients as having ‘low’ health literacy on the basis of low reading or numeracy skills, we fail to see the potential of many factors that influence their ability *to be literature about their own health condition.* A patient may be illiterate but have a very supportive partner that acts as a ‘surrogate’ reader, helping them understand the health information provided. Our findings demonstrate that categorising patients as having overall ‘high’ or ‘low’ health literacy is not useful in identify health literacy needs, or in developing interventions to address health literacy requirements within populations.

Most interventional health literacy studies to date have focused on reducing the putative reading level required to read and understand written information, and attempted to improve the composition or method of delivery of messages (including signage, brochures and instructional material) so that it is user-friendly [40, 41]. We found that strategies to improve written material were also important to participants in this study, however they formed only two of the 15 strategies identified by participants. Greater emphasis was placed on the importance of supportive relationships in responding to health literacy needs: between clinician and patient during the hospital stay, between the patient, carer and the hospital - ensuring carer’s involvement at times of key information provision; and between the hospital, the patient and the GP.

We previously found that being from a culturally and linguistically diverse background not only presents challenges for finding and understanding information, but also presents challenges around differing healthcare delivery contexts. Social support for health has been identified as a mediator of low health literacy in a number of studies [15, 42–44]. In our study, participants felt that encouraging patients to have carers to support them at key appointments may be of particular value for patients from a culturally and linguistically diverse background.

The reality of any hospital admission means that multiple health professionals will be involved in each patient’s care. Invariably, this means that patients are receiving, and are required to process, a large amount of complex and unfamiliar information from different professionals in a short period of time. This presents a challenge for hospitals and clinicians in ensuring each patient receives the information they require in order to manage their condition appropriately when they return home. Teach back is an evidence based intervention aimed at improving clinician-patient communication and patient knowledge retention [45–47]. Across the workshops, a number of strategies themed under “Quality communication during hospital stay”, and “A good discharge”, could be actioned through the application of teach back education. This method is suggested as a health literacy intervention by the Australian Commission on Safety and Quality in HealthCare [48], but as yet is not routinely used in the Australian and most other health care setting. Our research further supports adoption of this strategy in improving outcomes for patients in the hospital setting.

### Strengths and limitations

As the HLQ is a self-report measure of health literacy, it enables respondents to provide an indication of their self-perceived ability to achieve specific tasks related to managing their health (such as reading, understanding or completing medical forms). The benefit of this type of instrument is that it provides a rich data set on how socially connected patients feel engaged with their health, including feeling supported by health professionals and family, and how easy they find it to navigate their way around health systems. However, the disadvantage of self-report instruments are that higher and lower scores may reflect inflated or deflated views of competency [49].

The interventions designed for this population may or may not be appropriate for other hospital populations as patient needs may differ in other hospital settings. The Ophelia process should be tested in other populations, including at other hospitals and with more patient participants, to determine if there are common themes that arise.

All patients who participated in the workshops were over the age of 65. While we aimed to sample approximately one third under 65 (in line with response rates for the survey), on the day only individuals over the age of 65 attended. This may have been due to the workshops being run during working hours. This resulted in only seven patients (60% of those invited) attending the two patient workshops for patients. While we feel that by the end of the second workshop we had reached thematic saturation, a further workshop with more patients may have resulted in generation of new ideas. We overestimated the likelihood that patients would attend (having agreed to by phone) and in future we would oversample patient participants to allow for non-attendance.

A major strength of this study, using a two-step sequential explanatory design, is that the interventions were specifically designed to respond to the health literacy challenges of this population, capturing the whole spectrum of what we now know form an individual’s array of health literacy strengths and needs. The identification of strategies common across the workshops, verified by two independent researchers reviewing the transcripts and through member checks, suggests that these findings are valid for this hospital population. These findings are now in the process of being implemented, and future research in this hospital population will provide insight into whether these strategies can generate meaningful impacts, such as reducing potentially avoidable admissions or readmissions, and improving patient and staff experiences across the care continuum. Further research is required to identify whether these strategies are relevant to other patient populations.

## Conclusion

This study identified fifteen key strategies to manage the health literacy needs of a hospital population. We found that conducting a cross-sectional survey to identify a populations’ health literacy profile, and using cluster analysis to create user-friendly clinical vignettes, provided a locally driven, contextual approach to the co-design of interventions by patients and clinicians. Implementation and evaluation will assist to identify those strategies that have the maximum patient, clinician and/or organisational benefit. Further research may provide guidance into whether these interventions are applicable to the wider healthcare context.

## Additional file

Additional file 1: Cluster analysis, This additional file presents 3–13 and a 16 cluster solutions. The 8 cluster solution was the solution chosen for analysis as it presented the least amount of variation within clusters (based on the SD’s). (XLSX 48 kb)
